# The role of family communication patterns in intergenerational COVID-19 discussions and preventive behaviors: a social cognitive approach

**DOI:** 10.1186/s40359-023-01331-y

**Published:** 2023-09-26

**Authors:** Wanqi Gong, Li Crystal Jiang, Qin Guo, Fei Shen

**Affiliations:** 1 School of Journalism and Communication, Guangdong University of Foreign Studies, Guangzhou, China; 2 Department of Media and Communication, City University of Hong Kong, Hong Kong, 999077 China; 3 School of Humanities, Zhejiang University of Technology, Hangzhou, 310023 China

**Keywords:** Social cognitive theory, Family communication pattern, COVID-19, Intergenerational differences, Reverse socialization

## Abstract

**Background:**

This study explores and compares the influences of family communication patterns (conversation orientation and conformity orientation) on family discussion and preventive behaviors for older parents and their adult children in the context of the early COVID-19 outbreak.

**Methods:**

A total of 248 participants, including 117 parents and 131 adult children, participated in an online survey in February 2020. Participants reported family communication patterns, family discussions related to COVID-19, and their preventive behaviors.

**Results:**

Conversation orientation was positively associated with information sharing and scientific discussion for older parents and adult children. Our results revealed the differential influences of conformity orientation on older parents and adult children. Conformity orientation was positively associated with scientific discussion for older parents but was not significantly associated with any form of family discussion reported by adult children. There was a significant interaction effect of conversation orientation and conformity orientation on disputed communication within the family, suggesting that conflicts may arise in COVID-related discussions when parents and adult children value conversation and conformity. Scientific discussion was found to mediate the relationships between family communication patterns and preventive behaviors. The effects of scientific discussions were stronger for older parents than for adult children.

**Conclusions:**

Family communication patterns can be associated with preventive behaviors through different forms of family discussion about COVID-19. Conversation orientation is a strong facilitator for positive behavioral effects and scientific discussion is the most benign form of family health discussion. Health communication efforts should enhance the agency role of the family and motivate scientific discussion in health practices.

**Supplementary Information:**

The online version contains supplementary material available at 10.1186/s40359-023-01331-y.

## Background

The COVID-19 pandemic has exposed populations worldwide to unprecedented physical threats and psychological stress. As a novel virus, COVID-19 is highly contagious, and the infection outcomes are uncertain [1]. During the early outbreak stage, health professionals recommended preventive measures such as wearing facial masks, hand hygiene, and social distancing [2]. Many countries have implemented measures to prevent crowds from gathering and decrease transmission rates [3]. However, long-term quarantine can cause depression, anxiety, and other negative psychological symptoms [4, 5]. As a vulnerable group, older people have suffered exceptionally high levels of depression and isolation during the pandemic [4]. One study found that 24.5% of this group experienced psychological stress as the first wave of the pandemic spread across China [6].

A large body of research has found that elderly and middle-aged adults suffer more mental disorders than younger generations. Due to lower access rates to internet services, less use of mobile tools, and lower levels of e-health literacy, older adults are less likely to use the internet to seek health information than younger generations [7, 8]. When browsing COVID-related information on social media, elderly adults frequently report experiencing obsessive searching and information overload, further decreasing their self-efficacy toward COVID-19.9 However, the high volume of ambiguous COVID-19 information on social media has led to information overload and obsessive online information-searching behavior, likely to increase uncertainty, stress, and the intention to self-isolate [9, 10]. Scholars, therefore, call for greater agency and support in enhancing the self-efficacy and health literacy of middle-aged and elderly adults [11]. Family, media, community, and policymakers are significant stakeholders [12, 13]. Health campaigns and interventions have been widely implemented in the community and media for this group, but family-based actions for coping with COVID-19 are less emphasized in practices.

Families play a much more vital role in combat with COVID-19. Many people switched to the work-at-home model, and children spent significantly more time with their parents. Some stay with their families during lockdown and quarantine periods for care and support. The family has become a significant source of COVID-19 information during the quarantine period [14]. Therefore, the COVID-19 pandemic provides a unique context for understanding family influences on individuals’ preventive behaviors. In this study, we focused on China’s first outbreak of the COVID-19 pandemic. China executed lockdown measures from late January to March 2020, covering the Chinese New Year (CNY) holidays. There is a Chinese tradition of traveling back to one’s hometown and celebrating the CNY with the extended family. Hence most young adults stayed with their parents for the 2020 CNY during the lockdown. Lockdowns, fast-changing public agendas, and societal uncertainty uniquely characterize this period. In the first half of February, the agenda was dominated by uncertainty and fear of the virus. But as the pandemic progressed, the agenda switched to promoting behavioral compliance in the second half of February. Studies reported that when facing significant uncertainty about the coronavirus, the Chinese actively sought information and advised their family members on preventive behaviors [10, 15]. Television and social media were primary information sources during the national lockdown [16]. Older and young adults indicated differential preferences in information seeking. While older adults considered TV news more credible and authoritative, young adults relied more on social media platforms for timely information and help-seeking [17]. At the same time, they are more vulnerable to anxiety and depression caused by the proliferation of misinformation on and excessive use of social media during the COVID-19 pandemic [18].

The current study proposes to use the social-cognitive approach to examine how family communication may play a role in health information processing and preventive behaviors. Specifically, we integrate the social cognitive theory [19, 20] and the family communication patterns (FCP) theory [21] to argue that family communication patterns shape how the family shares and discusses health information and further influence the members’ preventive behaviors. Both theories claim that interpersonal processes can create specific environments to affect individual behaviors through reinforcement mechanisms. SCT formulates reciprocal determinism among personal, environmental, and behavioral factors [19]. When applied to explain health behaviors, SCT suggests that self-efficacy and specific goals can motivate performing health behaviors, and behavioral engagement further strengthens self-evaluations. Specific environments shape situated goals and activate the enactment of health behaviors; when individuals reflect on the consequences of their health behaviors, they may alter the environment to construct a more facilitating situation for health behaviors [20].

### Family communication patterns theory

Family provides a crucial environment for shaping individuals’ health attitudes and behaviors [22]. Abundant evidence suggests that family members are primary agents in health behavior change and decision-making. For example, they provide one another with instrumental and emotional support to deal with health crises [23]. However, fewer studies have considered family as an environment or a system that either energizes or dispirits health behaviors [24–26]. Thus, the study also incorporates the FCP Theory to postulate that families create their family communication environments, and such environments shape the communication behaviors among members and how well the families function [21].

Families differ significantly in parent-child interaction schemas and communication styles [27]. Mcleod and Chaffee [28] proposed two dimensions to capture family communication patterns. Socio-orientation signifies a family preference for harmonious relationships over ideas, while concept–orientation denotes a preference for ideas over relationships in the family. Ritchie and Fitzpatrick [29] refined this conceptualization and coined the two dimensions as conversation orientation and conformity orientation, which is also known for Revised Family Communication Patterns (RFCP) [21]. Conversation orientation refers to how parents encourage all family members to share feelings or beliefs on various topics. Conformity orientation labels the degree to which families emphasize the homogeneity of members’ beliefs, attitudes, and values [30, 31]. Families vary in their precedence and use of the two orientations to achieve agreements and complete family tasks [32].

FCP is theorized to affect cognitions and behaviors within the family, including members’ perceptions of the family environment and interpersonal relationships, children’s socialization, information exchange among members, and health decision-making [31, 33]. Previous research has shown that communicative outcomes differ markedly by orientation. A conversation orientation positively predicts satisfaction in parent-child communication, interpersonal communication skills, and disclosures of health issues to family members. On the other hand, a conformity orientation is likely to cause adverse communicative outcomes, such as family conflicts, psychological stress, and the concealment of sensitive health issues [24, 34, 35].

### Influence of FCP on Elders and Adult Children

The pattern of family communication is primarily shaped and controlled by parents. This forward perspective thus focused on how FCP influences children’s cognitions and behaviors [33]. Recent research has revealed a transmission of FCP within the dynamics of grandparent-parent-grandchildren relationships [36]. This implies that the FCP theory might encompass a wider scope than just parent-young child interaction. It is noteworthy that the influence can also be backward – FCP can influence parents’ knowledge, attitudes, and behaviors when parents are motivated to achieve agreements [37].

Previous studies found that children, especially adolescent and adult children, have reverse influences on their parents’ political opinions, new media adoption, and health behaviors [25, 38, 39]. Children can act as agents by discussing news and information with their parents, and such family discussion further shapes parents’ opinions and perceptions of FCP [38, 40]. Family studies have termed the backward effect as “reverse socialization,” which argues that intergenerational discussion is more important than the information exposure for older generations’ health and consumption behaviors [37, 41]. Albeit both forward and backward influences can occur in the family, most research mainly examines the effects of FCP on child development separately or vice versa [42, 43].

Contemporary China also provides a unique context for understanding the reciprocal influences because of the much stronger intergenerational influences documented in Chinese society [42].Therefore this study intends to investigate the reciprocity between parents and children and compares the influences of FCP on older parents and adult children in COVID-19 preventive behaviors. China’s extended family model and the one-child policy (1979–2015) have created close connections between parents and adult children [15]. For the above reasons, we consider the national lockdown during the 2020 CNY as an ideal context to examine the associations between family environments, family communication practices, and preventive behaviors.

FCP suggests that conversation orientation encourages family members to exchange their views on health issues, whereas conformity orientation places barriers to open discussion over health issues within the family [44]. According to Austin et al. 2018, family-based health information acquisition goes beyond information sharing. Scientific discussions that facilitate the interpretation and integration of health information and disputed discussions that pose barriers to information integration should also be considered [45]. Hence following the practices in previous research [46], we conceptualize open family discussion as content-specific forms relevant to the sharing, interpreting, and integrating of COVID-19 news. These forms include (a) frequent information sharing [44, 47], (b) more scientific discussion that requires critical interpretation and integration of health information [46], and (c) fewer disputes in family discussions [45]. Conversation orientation is expected to create an encouraging environment where parents and adult children are open to exchanging thoughts and emotions. By contrast, conformity orientation may restrict information exchange and motivate conflict avoidance to achieve intergenerational agreement. Therefore, we propose the following hypotheses:

#### H1

Conversation orientation is positively related to the older parents’ (a) information sharing and (b) scientific discussion with their adult children, but (c) negatively related to the parents’ disputed discussion with their adult children.

#### H2

Conformity orientation is negatively related to the older parents’ (a) information sharing and (b) scientific discussion with their adult children, but (c) positively related to the parents’ disputed discussion with their adult children.

#### H3

Conversation orientation is positively related to the adult children’s (a) information sharing and (b) scientific discussion with their parents, but (c) negatively related to the adult children’s disputed discussion with their parents.

#### H4

Conformity orientation is negatively related to the adult children’s (a) information sharing and (b) scientific discussion with their parents, but (c) positively related to the adult children’s disputed discussion with their parents.

It is noteworthy that conversation and conformity orientations are not orthogonal dimensions [29]. Families can score low on conformity and conversation orientation when parents and children have little interaction. Family may also score high on both dimensions when parents follow children’s ideas to achieve intergenerational agreement. However, a meta-analysis of 32 empirical studies suggests that the two orientations are inversely correlated [33]. Follow-up studies also found significant interaction effects of conversation and conformity orientation on family communication outcomes [35, 48]. Thus, we asked the following question to probe for the interaction effect of two dimensions.

#### RQ1

Is there any interaction effect of conversation orientation and conformity orientation on family discussion related to COVID-19 for older parents and adult children?

### Family Communication and preventive behaviors

SCT and FCP both theorize interpersonal processes as mediating mechanisms linking environmental factors to behavioral outcomes. In particular, the interactions between family members can encourage or discourage certain behaviors by altering individuals’ goal-setting and outcome expectations [19]. Family communication is theorized to mediate the socialization process through behavioral modeling and reinforcement [21, 49]. Empirical evidence supports that parent-child communication mediates the parental influences on child development and young adults’ health behaviors [24, 34, 50].

Although there is scarce research on the role of family communication in reverse socialization in health behaviors, previous studies show that intergeneration learning may impact older generations’ perceptions and behaviors in environmental education and health promotion [12, 13]. Older people have benefitted from the knowledge of family members to learn about the virus and overcome their mental stress during the pandemic [25, 51, 52]. Adult children can help their parents to navigate the constant stream of COVID-19 information, provide health advice and seek help on the internet [10, 15]. Intergenerational communication may also alleviate older adults’ loneliness and psychological stress, enhancing their health and encouraging protective behaviors [53]. Thus, we propose the following hypothesis:

#### H5

(a) information sharing, (b) scientific discussion, and (c) disputed discussion mediate the relationship between FCP and preventive behaviors.

It is noteworthy that some research reveals generational differences in family communication and intergenerational relationships [42]. This study also explores whether the forward and backward effects differ by family members:

#### RQ2

Do the associations between FCP, family discussion, and preventive behaviors significantly differ by older parents and adult children?

## Methods

### Participants

A total of 248 Chinese participants were recruited for the study, including 131 adult children and 117 parents. As shown in Table 1, the adult child participants were between 20 and 24 (*M* = 21.14, *SD* = 0.88), and 82.4% were female college students. The parent participants were between 40 and 63 years (*M* = 49.28, *SD* = 3.83), and 56.4% were females. The parent participants’ level of education was relatively low: 36.5% had attended middle school or below, 33.3% high school or equivalent, 14.5% junior college, and 16.3% were university graduates. Nearly half of the parent participants reported a monthly income of less than 5,000 RMB (49.6%), 31.6% between 5,000 and 9,999 RMB, 10.3% between 10,000 and 19,999 RMB, and 8.5% at least 20,000 RMB.

**Table 1:** Characteristics of the participants

Older parents	
Female, *N* (%)	66 (56.4)
Age, *M* (*SD*), [range]	49.28 (3.83), [40 − 63]
**Educational level**, ***N*(*%*)**	
Primary school and/or below	9 (7.7)
Middle school	33 (28.2)
High school/vocational qualifications or equivalent	39 (33.3)
Junior college	17 (14.5)
Bachelor degree	16 (13.7)
Master degree or above	3 (2.6)
**Monthly income**	
Up to ¥2,999	24 (20.5)
¥3,000 to ¥4,999	34 (29.1)
¥5,000 to ¥9,999	37 (31.6)
¥10,000 to ¥19,999	12 (10.3)
More than ¥20,000	10 (8.5)
**Adult children**	
Female, *N* (%)	108 (82.4)
Age, *M* (*SD*), [range]	21.14 (0.88), [20 − 24]
*M, mean; SD, stand deviation.*	

### Procedure

The study was approved by the authors’ institutional review board, and informed consent was obtained from the participants. The data were collected between February 10 and 24, 2020, when the Chinese government implemented the social distancing policy. College students from two universities were asked to invite their parents to participate in an online survey. Extra course credit was given as an incentive for participation. The self-report survey questionnaires for parents and their adult children were identical in content, and each contained five parts: (a) Family communication patterns; (b) Family discussion topics related to COVID-19; (c) Preventive behaviors related to COVID-19; and (d) Basic demographic information.

### Measures

*Family communication patterns* were measured by Ritchie’s RFCP scale [21]. This scale is based on FCP but provides a better label and operationalizes the underlying dimensions of the family communication environment [54]. The instrument required parents and adult children to rate their communication patterns within the family. The scale consists of two dimensions: conversation orientation and conformity orientation, each comprising five items. The RFCP scale has been translated and validated in Chinese for adolescent research [55]. We utilized the Chinese version of the RFCP scale and ensured the translation was consistent with the Chinese context.

*Family discussion over COVID-19* scale was specifically created for this study. It was designed by drawing upon prevalent themes that emerged from extensive discussions on social media platforms and in interpersonal conversations during that period [56, 57]. The scale included three dimensions with varying valence and engagement levels: COVID-related information sharing, scientific discussion, and disputed communication. COVID-related *information sharing*, the exchange of information within the family, was measured by three items. *Scientific discussion*, the interpretation and integration of COVID-related scientific knowledge in the family, was measured by five items. Finally, *disputed communication*, the conflicts and disagreements over COVID-related topics among family members, was measured by two items.

*COVID-19 preventive behavior scale* was developed based on the Health Education Manual on COVID-19 published by the Chinese National Health Commission [58]. This manual covers various aspects, including COVID-related knowledge, personal prevention, home prevention, workplace prevention, and more. It was intended for both the general public and professional organizations at all levels as a reference and resource. Based on the manual, we included two dimensions in our preventive behavior measures: social distancing and cleanliness control. *Social distancing* was measured by three items that described the degree to which people maintain social distancing from others. *Cleanliness control* was measured by two items that respectively assessed the extent to which people maintain regular handwashing and household hygiene.

In the study, all the items were rated on a 5-point Likert scale ranging from 1 = *never/totally disagree* to 5 = *most frequently/totally agree*. After evaluating the validity and reliability of the scales, the results were averaged to create composite scores for statistical analysis. The core measurements for the study are shown in Table 2; The complete questionnaire is attached in Appendix I.

**Table 2:** Items comprising the constructs in the measurements

Construct	Items	Factor loading	Composite reliability	AVE
**Conversation orientation**			0.877	0.642
	1. Family members always share ideas	0.683		
	2. Family members always talk about everyday life	0.786		
	3. Family members are always willing to chat	0.750		
	4. Family members chat frequently	0.828		
	5. Family members are always talking about future	0.781		
**Conformity orientation**			0.803	0.506
	1. Parents demand obedience from children	0.602		
	2. Parents make family decisions	0.551		
	3. Parents angry over disagreements	0.788		
	4. Parents demand compliance with rules	0.813		
	5. Parents always say, “when you grow up you will understand”	0.517		
**Information sharing**			0.803	0.577
	1. I share information and knowledge related to COVID-19 with family members (e.g., infection, transmission)	0.835		
	2. I share news regarding the severity of COVID-19 with family members	0.702		
	3. I share news regarding treatment with family members	0.781		
**Scientific discussion**			0.884	0.657
	1. My family and I discuss the scope of the pandemic	0.554		
	2. My family and I talk about the consequences of infection	0.681		
	3. My family and I discuss about the measure of social distancing	0.849		
	4. My family and I discuss the measure of wearing a mask	0.910		
	5. My family and I discuss the measure of washing hands regularly	0.892		
**Disputed communication**			0.822	0.698
	1. My family and I had disagreements over COVID-19 prevention	0.813		
	2. My family and I had disagreements over COVID-19 treatments	0.798		
**Social distancing**			0.821	0.612
	1. I wore a mask when going out after the outbreak	0.477		
	2. I avoided gathering after the outbreak	0.847		
	3. I reduced the frequency of outdoor activity after the outbreak	0.788		
**Cleanliness control**			0.645	0.476
	1. I often cleaned the households after the outbreak	0.625		
	2. I often wash my hands after the outbreak	0.717		

### Statistical analysis

**Table 3:** Summary of the measurement scales

Scales	Older parents		Adult children	
	*M*, (*SD*)	Reliability	*M*, (*SD*)	Reliability
Conversation orientation	3.656 (0.725)	0.895	3.218 (0.823)	0.903
Conformity orientation	3.164 (0.750)	0.817	3.357 (0.756)	0.795
Information sharing	3.900 (0.720)	0.804	3.852 (0.708)	0.809
Scientific discussion	4.065 (0.677)	0.884	4.043 (0.726)	0.907
Disputed communication	2.145 (0.996)	0.752	1.985 (0.992)	0.839
Social distancing	4.615 (0.479)	0.704	4.768 (0.453)	0.813
Cleanliness control	4.150 (0.680)	0.594	3.859 (0.768)	0.678

M, mean; SD, stand deviation

**Fig. 1:**
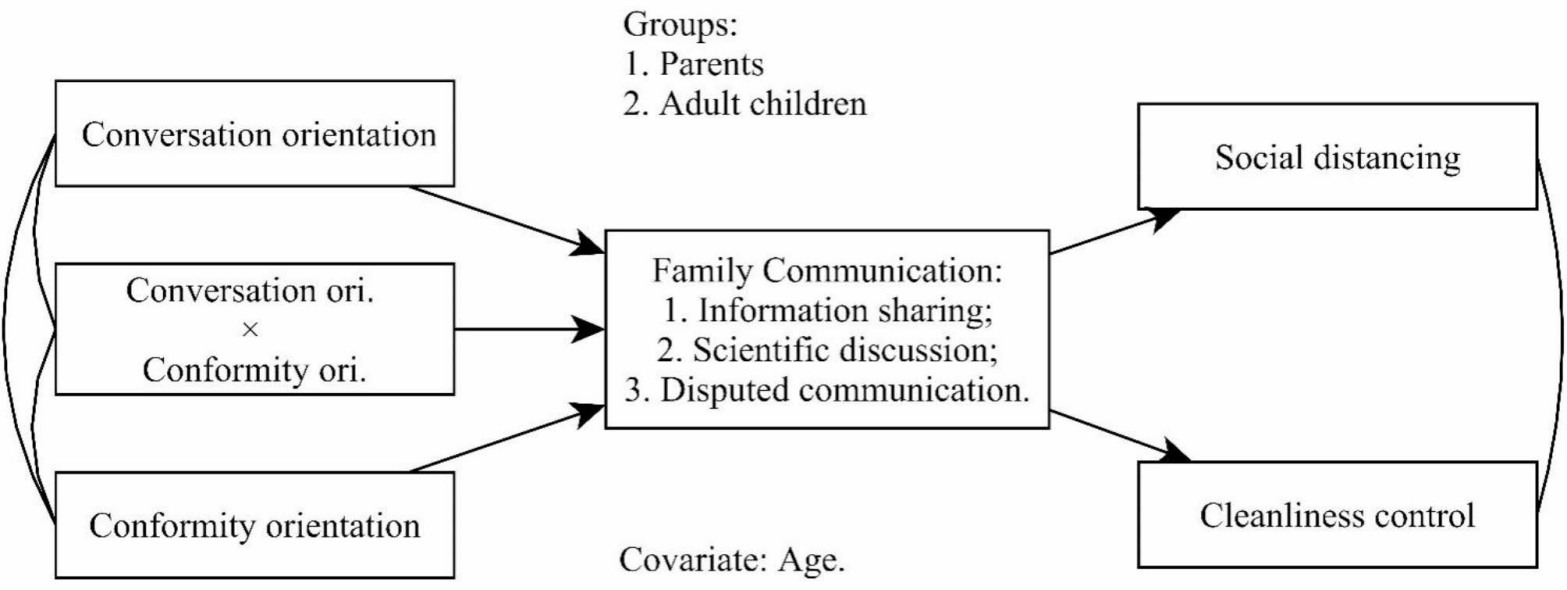
The hypothesized mediation model

An Exploratory factor analysis (EFA) and confirmatory factor analysis (CFA) were conducted to examine the convergent and discriminant validity of the measures (see Table 2 for measurement evaluations). A multigroup mediation path model was used to test the effects of family discussion on the relationships between family communication patterns and preventive behaviors and to estimate differences between older parents and their adult children on these issues. The dataset was divided into two sub-samples representing the older parents’ group and the adult children’s group, and critical ratios for difference analysis were calculated. In the constructed model (Fig. 1), two FCP variables and their interaction term were considered independent variables, three forms of family discussions as mediators, two preventive behaviors as dependent variables, and the participant’ age as a covariate. Two FCP variables were grand mean centered before computing the interaction term. All direct and indirect paths between FCP variables, FCP interaction, family discussion, and preventative behaviors were first connected. We also correlated conversation orientation and conformity orientation, and social distancing and cleanliness control in the model. We also built several alternatives to identify the best-fitting model for the data.

Kline recommends a minimum sample-size-to-parameters ratio of 10:1 for a path model [59]. This means that a minimum sample size of 10 responses is required for each parameter to ensure statistical precision. The path model in this study has 19 parameters. Thus, a participant count of 248 for the study is deemed appropriate and acceptable.

According to Kenny [60], the path model is considered to fit the data well if the comparative fit index (CFI) and Tucker Lewis index (TLI) values exceed 0.95, root mean square error of approximation (RMSEA) and standardized root mean square residual (SRMR) are lower than 0.08, and p of close fit (PCLOSE) is larger than 0.05. Akaike Information Criterion (AIC) was used to evaluate the nested path models. Usually, a smaller AIC indicates a better fit for that data. The internal consistency of the measurement scales was calculated with Cronbach’s Alpha; a coefficient larger than 0.7 was considered acceptable. The Spearman-Brown coefficient was used to test the reliability of the two-item scales [61]. If the critical ratio (*z*-score) of the difference between the path coefficients was larger than 1.96 or less than − 1.96, then the difference between the paths was significant at *p* = 0.05.

The EFA was performed using SPSS 26.0, CFA and the mediation model were built using AMOS 24.0. The mediation effects were calculated using Gaskin’s AMOS plugin [62], and bootstrapping with a 95% confidence interval was used in the study (N = 2000).

### Factor analyses and model testing

An EFA with principal components method with varimax rotation was performed to examine the dimensionality of each measure. The results indicated that the 25 items comprised seven factors that explained 70.677% of the total variance (factor loadings ranged between 0.593 and 0.901).

A CFA model was constructed to assess the convergent and discriminant validity of the primary constructs. The CFA model displayed a goodness of fit for our data, as indicated by a χ^2^(251) = 599.362, CFI = 0.940, TLI = 0.928, RMSEA = 0.053, SRMR = 0.0489; PCLOSE = 0.159. The items were loaded as expected, with factor loadings ranging from 0.550 to 0.908 (Table 2). We also separately performed the analysis on parents’ responses and adult children’s responses to ensure equivalent factor structures (Table 3). The results also indicated acceptable convergent validity for most constructs (composite reliability larger than 0.70; AVE larger than 0.50) except for the cleanliness control (composite reliability = 0.645; AVE = 0.476). Considering the scale of cleanliness control consisted of only two items, the result is acceptable [61]. Discriminant validity was assessed using the Fornell–Larcker criterion. Table 4 suggests that the square root of the AVE of each construct was larger than its correlation with other constructs, demonstrating satisfactory discriminant validity.

**Table 4:** Discriminant validity assessment using Fornell–Larcker criterion

Construct	1	2	3	4	5	6	7
(1) Conversation orientation	**0.801**						
(2) Conformity orientation	-0.048	**0.711**					
(3) Information sharing	0.544	0.087	**0.760**				
(4) Scientific discussion	0.355	0.077	0.642	**0.811**			
(5) Disputed communication	0.191	0.219	0.274	0.225	**0.835**		
(6) Social distancing	0.098	0.000	0.148	0.188	-0.041	**0.782**	
(7) Cleanliness control	0.368	0.004	0.448	0.394	0.196	0.531	**0.690**

Statistical power was evaluated using the Post-hoc Statistical Power Calculator [63]. For both dependent variables, social distancing and cleanliness control, the observed statistical power was 0.99. This result indicates that the model provides enough statistical power to test the proposed hypotheses.

Table 5 presents the model selection results. Model 2 had the smallest AIC value among all the models, but Model 1 and Model 2 had a similar model fit (∆AIC = 19.647). Thus, we selected Model 1 as the best-fitting model, considering that it captured more nuance than Model 2; χ^2^(14) = 19.643, *p* = 0.142; CFI = 0.986, TLI = 0.927, RMSEA = 0.040, SRMR = 0.0334; PCLOSE = 0.611.

**Table 5:** Model fit indices for alternative models

Model construction	AIC
Model 1: the selected model	207.643
Model 2: based on model 1, direct paths between FCP, FCP interaction and preventative behaviors were removed	187.996
Model 3: based on model 1, the correlations between conversation orientation, conformity orientation and interaction term, and between social distancing and cleanliness control were removed	309.414
Model 4: based on model 2, all correlation paths were removed	289.819

FCP, family communication patterns; AIC, Akaike information criterion

## Results

### Relationships between FCP and family discussion

H1 to H2 predicted the relationships between FCP and family discussion related to COVID for parents. As shown in Table 6, the parents scoring high in conversation orientation were more likely to share COVID-related information (β = 0.492, *p* < 0.001) and engage in scientific discussion (β = 0.462, *p* < 0.001) with their adult children. But their conversation orientation was not associated with disputed communication (*p* = 0.373). Therefore, H1 was partially supported.

**Table 6:** Path analysis results and z-score comparison (Bootstrap N = 2000)

	Older parent-subgroup			Adult child-subgroup			*z*-score
	B	β	*p*	B	β	*p*	
Conversation ori. → Information sharing	0.489	0.492	< 0.001	0.370	0.430	< 0.001	−1.115
Conversation ori. → Scientific discussion	0.432	0.462	< 0.001	0.297	0.336	< 0.001	−1.265
Conversation ori. → Disputed communication	0.108	0.078	0.373	0.369	0.306	< 0.001	1.648
Conversation ori. → Social distancing	0.028	0.042	0.660	0.007	0.013	0.899	−0.243
Conversation ori. → Cleanliness control	0.017	0.018	0.842	0.026	0.028	0.774	0.068
Conformity ori. → Information sharing	0.044	0.046	0.640	0.147	0.156	0.057	0.839
Conformity ori. → Scientific discussion	0.198	0.219	0.026	0.076	0.078	0.357	−1.009
Conformity ori. → Disputed communication	0.204	0.154	0.152	0.152	0.116	0.174	−0.286
Conformity ori. → Social distancing	−0.026	−0.041	0.691	−0.005	−0.009	0.924	0.244
Conformity ori. → Cleanliness control	−0.126	−0.138	0.161	−0.012	−0.012	0.888	0.910
Conversation ori. × Conformity ori. → Information sharing	−0.054	−0.052	0.596	−0.058	−0.053	0.507	−0.031
Conversation ori. × Conformity ori. → Scientific discussion	−0.042	−0.043	0.664	−0.105	−0.092	0.265	−0.467
Conversation ori. × Conformity ori. → Disputed communication	0.307	0.214	0.046	0.271	0.175	0.034	−0.180
Conversation ori. × Conformity ori. → Social distancing	0.018	0.026	0.801	−0.065	−0.093	0.296	−0.084
Conversation ori. × Conformity ori. → Cleanliness control	0.139	0.142	0.146	−0.035	−0.029	0.723	−1.267
Information sharing → Social distancing	0.011	0.016	0.888	0.036	0.057	0.608	0.241
Information sharing → Cleanliness control	0.211	0.222	0.047	0.012	0.011	0.913	−1.290
Scientific discussion → Social distancing	0.269	0.377	0.001	0.017	0.027	0.798	−2.368*
Scientific discussion → Cleanliness control	0.348	0.344	0.002	0.320	0.305	0.002	−0.184
Disputed communication → Social distancing	−0.044	−0.091	0.287	0.037	0.082	0.376	1.377
Disputed communication → Cleanliness control	−0.024	−0.035	0.668	0.125	0.163	0.059	1.715
Age → Conversation ori.	−0.014	−0.076	0.409	−0.165	−0.175	0.043	−1.810
Age → Conformity ori.	0.002	0.011	0.881	0.123	0.143	0.099	1.590
Age → Social distancing	−0.031	−0.248	0.002	0.019	0.036	0.681	1.068
Age → Cleanliness control	−0.017	−0.095	0.222	−0.033	−0.038	0.650	−0.213
Conversation ori. ↔ Conformity ori.	*r* = − 0.056		0.540	*r* = − 0.218		0.008	
Conversation ori. ↔ Conversation ori. × Conformity ori.	*r* = − 0.090		0.505	*r* = − 0.005		0.954	
Conformity ori. ↔ Conversation ori. × Conformity ori.	*r* = 0.577		0.002	*r* = − 0.079		0.553	
Social distancing ↔ Cleanliness control	*r* = 0.496		< 0.001	*r* = 0.455		< 0.001	

†*p* < 0.10, *p < 0.05, **p < 0.01; Model fits: χ^2^_(14)_ = 19.643, *p* = 0.142; CFI = 0.986, TLI = 0.927, RMSEA = 0.040, SRMR = 0.0334; PCLOSE = 0.611.

The parents scoring high in conformity orientation were more likely to have scientific discussions (β = 0.219, *p* = 0.026) with their adult children. But conformity orientation was not related to their COVID-related information sharing (*p* = 0.640) and disputed conversations with their adult children (*p* = 0.152). H2 was, therefore, partially supported.

H3 and H4 hypothesized the relationships between FCP and family discussion for adult children. In full support of H3, the adult children scoring high in conversation orientation were more likely to share COVID-related information (β = 0.430, *p* < 0.001), engage in scientific discussion (β = 0.336, *p* < 0.001), and have disputed communication (β = 0.306, *p* < 0.001) with their parents. Their conformity orientation was not associated with any forms of COVID-related family discussion (for, information sharing, *p* = 0.057; for scientific discussion, *p* = 0.357; for disputed communication, *p* = 0.174). Therefore, H4 was not supported.

### The effect of conversation and conformity interaction term

RQ1 examined the effect of FCP interaction (conversation orientation × conformity orientation) on family discussion.

As shown in Table 6, the interaction between conversation orientation and conformity orientation was significant for disputed communication within the family for older parents (β = 0.214, *p* = 0.046) and adult children (β = 0.175, *p* = 0.034). This means that when parents and adult children reported high conversation orientation and high conformity orientation, they were more likely to have disagreements and conflicts in their COVID-related discussions. The interaction effect was insignificant for information sharing (for parents, *p* = 0.596; for adult children, *p* = 0.507) and scientific discussion (for parents, *p* = 0.664; for adult children, *p* = 0.265). Likely, the interaction had no effect on social distancing (for parents, *p* = 0.801; for adult children, *p* = 0.296) and on cleanliness control (for parents, *p* = 0.146; for adult children, *p* = 0.723).

As shown in Table 7, three forms of family discussion did not mediate the relationships between FCP interaction and preventive behaviors for older parents and their adult children.

**Table 7:** Mediation paths results (Bootstrap N = 2000)

	Older parent-subgroup			Adult child-subgroup		
Paths	B	95% CI	*p*	B	95% CI	*p*
Conversation ori. → Social distancing						
Conversation ori. → Information sharing → Social distancing	0.005	[− 0.078, 0.085]	0.856	0.013	[− 0.029, 0.063]	0.525
Conversation ori. → Scientific discussion → Social distancing	0.116	[0.041, 0.219]	< 0.001	0.005	[− 0.059, 0.044]	0.856
Conversation ori. → Disputed communication → Social distancing	−0.005	[− 0.040, 0.006]	0.314	0.014	[− 0.008, 0.058]	0.187
**Conversation ori. × Cleanliness control**						
Conversation ori. → Information sharing → Cleanliness control	0.103	[− 0.015, 0.226]	0.090	0.004	[− 0.081, 0.097]	0.837
Conversation ori. → Scientific discussion → Cleanliness control	0.150	[0.038, 0.308]	0.008	0.095	[0.028, 0.210]	0.004
Conversation ori. → Disputed communication → Cleanliness control	−0.003	[− 0.046, 0.011]	0.510	0.046	[0.005, 0.112]	0.035
**Conformity ori. × Social distancing**						
Conformity ori. → Information sharing → Social distancing	0.000	[− 0.015, 0.024]	0.873	0.005	[− 0.008, 0.046]	0.375
Conformity ori. → Scientific discussion → Social distancing	0.053	[0.001, 0.134]	0.046	0.001	[− 0.014, 0.027]	0.578
Conformity ori. → Disputed communication → Social distancing	−0.009	[− 0.052, 0.006]	0.207	0.006	[− 0.004, 0.048]	0.231
**Conformity ori. × Cleanliness control**						
Conformity ori. → Information sharing → Cleanliness control	0.009	[− 0.029, 0.088]	0.504	0.002	[− 0.030, 0.052]	0.677
Conformity ori. → Scientific discussion → Cleanliness control	0.069	[0.007, 0.183]	0.032	0.024	[− 0.025, 0.099]	0.287
Conformity ori. → Disputed communication → Cleanliness control	−0.005	[− 0.055, 0.015]	0.495	0.019	[− 0.008, 0.078]	0.161
**Conversation ori. × Conformity ori. × Social distancing**						
Conversation ori. **×** Conformity ori. → Information sharing → Social distancing	−0.001	[− 0.025, 0.015]	0.790	0.000	[− 0.031, 0.007]	0.447
Conversation ori. **×** Conformity ori. → Scientific discussion → Social distancing	−0.011	[− 0.076, 0.039]	0.598	0.000	[− 0.032, 0.020]	0.648
Conversation ori. **×** Conformity ori. → Disputed communication → Social distancing	−0.014	[− 0.069, 0.008]	0.203	0.010	[− 0.005, 0.050]	0.171
**Conversation ori. × Conformity ori. × Cleanliness control**						
Conversation ori. **×** Conformity ori. → Information sharing → Cleanliness control	−0.011	[− 0.083, 0.028]	0.464	0.000	[− 0.044, 0.020]	0.611
Conversation ori. **×** Conformity ori. → Scientific discussion → Cleanliness control	−0.015	[− 0.087, 0.050]	0.508	−0.030	[− 0.141, 0.018]	0.190
Conversation ori. **×** Conformity ori. → Disputed communication → Cleanliness control	−0.007	[− 0.072, 0.025]	0.505	0.034	[− 0.001, 0.094]	0.056

95% CI, 95% confidence interval

### Mediation effects of family discussion on the relationships between FCP and preventive behaviors

H5 predicted the mediation effects of family discussion on the relationships between FCP and preventive behaviors. As indicated by Table 7, for parents, scientific discussion mediated the relationships between FCP (both conversation and conformity orientations) and preventive behaviors (both social distancing and cleanliness control). Specifically, bootstrapping results showed significant indirect effects of conversation orientation on social distancing (B = 0.116, 95% CI [0.041, 0.219], *p* < 0.001) and cleanliness control (B = 0.150, 95% CI [0.038, 0.308], *p* = 0.008) via scientific discussion. Likewise, there were significant indirect effects of conformity orientation on social distancing (B = 0.053, 95% CI [0.001, 0.134], *p* = 0.046) and cleanliness control (B = 0.069, 95% CI [0.007, 0.183], *p* = 0.032) via scientific discussion. Information sharing and disputed communication did not appear to mediate the relationships between FCP and preventive behaviors for parents (see Table 7).

The mediation effects for adult children were quite scattered. Similar to the results revealed for older parents, scientific discussion mediated the relationship between conversation orientation and cleanliness control (B = 0.095, 95% CI [0.028, 0.210], *p* = 0.004). Disputed communication also mediated the relationship between conversation orientation and cleanliness control (B = 0.046, 95% CI [0.005, 0.112], *p* = 0.035). Information sharing was not a significant mediator between FCP and preventive behaviors, and the data supported no other mediating effects. These results indicate limited support for H5.

### Intergenerational differences in the effects of FCP on family discussion and preventive behaviors

RQ2 addressed group differences in the associations between FCP, family discussion, and preventive behaviors. Table 6 presents the comparison of critical ratios across two sub-groups. The effect of scientific discussion on social distancing among older parents (β = 0.377) was more significant than that among adult children (β = 0.027; *z* = − 2.311, *p* < 0.050), which suggests that older parents benefited more from family-based scientific discussion than adult children.

## Discussion

Family is a crucial environmental factor influencing people’s health attitudes and behaviors. Drawing on social cognitive theory and FCP, we examined how family communication traits shape how older Chinese parents and their adult children communicate about and cope with the COVID-19 pandemic. We investigated three forms of family discussion, including COVID-19 (information sharing, scientific discussion, and disputed communication), and tested them as mediators between FCP and preventive behaviors. We also compared the associations between FCP, family discussion, and preventive behaviors across parents and adult children. Our results suggest that conversation orientation reported by parents and adult children was associated with more frequent information sharing and scientific discussion within the family. Adult children’s conversation orientation was associated with more disputed communication regarding COVID-19, while older parents’ conformity orientation was associated with more frequent scientific discussion. Scientific discussion mediated the relationships between two FCP dimensions and preventive behaviors for older parents. There was a significant interaction effect between conversation orientation and conformity orientation on disputed communication for both groups, revealing that high conversation orientation and high conformity orientation are likely to create disagreements and conflicts in COVID-related discussions. Most associations did not differ across the two groups, except that scientific discussion had a more substantial effect on social distancing for older parents than for adult children.

### Theoretical implications

Our analysis provides empirical evidence for the influence of family communication on family members’ health behaviors. Aligned with previous studies [24, 31, 34], conversation orientation is connected with open discussion and active prevention for older parents and their adult children. When family members feel comfortable about family dialogues, they are more likely to share health information and reveal their thoughts on health topics. Interestingly, conversation orientation is positively associated with disputed communication for adult children. The dialogue-oriented environment makes children less afraid to dispute with their parents.

Notably, although hypothesized as a negative predictor, conformity orientation was not negatively associated with communication behaviors. Our results even indicated that conformity orientation was positively associated with scientific discussion reported by older parents. When conformity is desired within the family, parents can be motivated to discuss health topics with adult children to achieve family consensus. Such findings enrich our understanding of “reverse socialization” or backward influence in intergenerational communication. Scholars often claim that conversation orientation nurtures open discussion and better communication outcomes [38, 39], but as revealed in our results, conformity orientation can create compliance for older parents to engage in scientific discussion. On the other hand, conversation orientation is not always associated with positive outcomes. When high conversation and conformity orientation are desired, family members could experience more turbulence and disputes. These findings point to the potential of differential perceptions of power dynamics in family communication. Parents generally hold power to establish a strong conformity orientation within the family and use this to establish the correct guidance for their adult children via family discussions about preventive measures. They construct the family discussion as a series of parent-led educational activities. By contrast, the positive association between conversation orientation and information sharing reported by young adults may reflect their attempts to negotiate power relations within the family. Young adults are motivated to show that they can influence their parents by sharing important information with them.

Of the three forms of family discussion, scientific discussion demonstrated the most salient effects in the COVID-19 context. Scientific discussion mediated the relationships between FCP and preventive behaviors for both generations. By contrast, although conversation orientation is positively associated with information sharing, exposure to health information is not associated with preventive behaviors. These findings reveal more nuances about how effective family communication affects health attitudes and behaviors. Sharing COVID-19 information is not enough to motivate preventive behaviors. The behavioral effects depend on how family members interpret and integrate health information [46]. Family members, especially the older generations, become capable agents upon internalizing family discussions [25, 38–40].

### Practical implications

Our results identified scientific discussion as a particularly beneficial form of family discussion for older parents. While most associations were equivalent for both groups, scientific discussion had a stronger association with social distancing for older parents than for adult children. We recommend that health promotion targeted at older adults should go beyond information sharing. Health communication practitioners explore multiple ways to incorporate and enhance scientific discussion with older people. Specifically, guidance could be provided to young adults on how to discuss health issues with their parents. Both young adults and their parents should be informed that intergenerational differences are common and stem from differences in the sources of information accessed by each generation. Also useful is the knowledge that disagreements and disputes can be solved or reconciled through a conversation orientation. Both generations should be advised to take this orientation and engage in rational discussions of the scientific facts, rather than letting their predispositions (e.g., to achieve conformity, to avoid disputes) predefine the talk.

Our findings confirmed the importance of families in implementing effective coping strategies for COVID-19 as health policies and recommended practices kept changing during the national lockdown in China. Families with high conversation orientation benefit the most from openly discussing COVID-19 and collectively performing the preventive measures. As we attempt to help families recover from the pandemic and build long-term resilience for future health crises, it is essential to cultivate conversation orientation as a means to leverage the power of family. To accomplish this, parents and adult children should be informed about the forward and backward perspectives in family communication and health decision-making. Both parties should develop an awareness of healthy power dynamics in family communication. This is a necessary lesson for the Chinese as people in general view family discussions as shared missions instead of power negotiation.

### Limitations and Future Research

This study has several limitations that future research should address. The primary limitation of this study is that our sample size was small. Our data collection window was short, and we experienced difficulties in recruiting parent-adult child pairs. The small sample did not allow for more advanced statistical analysis (e.g., full structural modeling). The small sample size may cause power problems - we cannot rule out the possibility that some hypothesized paths were not significant due to lack of power. Another limitation concerns the nature of the sample, which consisted of college students and their parents. The parents were predominantly middle-aged adults and most respondents of both generations reported higher levels of education, meaning that our findings may not be generalizable to the wider population.

Another area for improvement of the study is the use of two-item scale for cleanliness control, which may compromise the validity of the measures. Although using two items to measure cleanliness control may seem limited, both items have face validity as their wording and content align with what was recommended by the authority in February 2020.

The findings may also be specific to the cultural context of this research, with the possibility that the collectivistic background produced a particularly strong effect of family communication patterns on intergenerational health communication and preventive behaviors. Future studies should therefore test the proposed framework in individualistic contexts or conduct cross-cultural comparisons to establish its explanatory reach. Future research may also benefit from employing independent estimates or inventory-based measures to control the potential bias caused by interpretive schemes.

Third, the cross-sectional design did not allow us to establish causality in our findings. We cannot rule out the possibility that our proposed framework operates differently (e.g., information sharing and scientific discussion shape or redefine family communication patterns). In addition, several recent studies have pointed out that cross-sectional data may be biased when applying mediation analysis [63], and therefore our results may be affected. We thus call for future research that examines causal relationships through experimental or longitudinal designs.

## Conclusion

The present study offers empirical evidence supporting the positive role of conversation orientation in shaping preventive behaviors in the COVID-19 context. Scientific discussion between older parents and their adult children mediated the relationship between family communication patterns and preventive behaviors. Some intergenerational differences are also noted, whereby scientific discussion plays a more substantial role for older parents than adult children. Health interventions should utilize family communication and pay critical attention to scientific discussion within the family to facilitate recovery and build resilience.

### Electronic supplementary material

Below is the link to the electronic supplementary material.

Supplementary Material 1

## Data Availability

The datasets used and/or analyzed during the current study available from the corresponding author on reasonable request.
