# Supplementary material for: The role of family communication patterns in intergenerational COVID-19 discussions and preventive behaviors: a social cognitive approach
Source: BMC Psychol. 2023 Sep 26;11:290. doi: 10.1186/s40359-023-01331-y (PMC10523603; doi:10.1186/s40359-023-01331-y)
Supplement: Supplementary file 1 — Supplementary Material 1 [file 40359_2023_1331_MOESM1_ESM.docx]

**Appendix I**

**Questionnaire on Self-Protection and Family Communication Regarding the COVID-19 Pandemic**

In early 2020, our country experienced an outbreak of the COVID-19 pandemic, with a widespread impact. We hope to gather your opinions regarding the pandemic through this questionnaire. Please answer the following questions based on the information you have encountered and your views on the pandemic-related issues.

Your participation is very important to us, and the answers will be kept strictly confidential and used only for academic research, without any impact on your privacy. Thank you very much for your participation!

1. After learning about the pandemic, have your regular behaviors changed in any way? *(Measured with a 5-point Likert scale ranging from 1= totally disagree to 5= totally agree)*
2. I wore a mask when going out after the outbreak.
3. I avoided gathering after the outbreak.
4. I reduced the frequency of outdoor activity after the outbreak.
5. I often cleaned the households after the outbreak.
6. I often wash my hands after the outbreak.
7. We would like to understand your usual communication patterns with your family members. Could you please indicate to what extent your communication with your family members (parents/adult children) aligns with the following descriptions?

*(Measured with a 5-point Likert scale ranging from 1= totally disagree to 5= totally agree)*

1. Family members always share ideas.
2. Family members always talk about everyday life.
3. Family members are always willing to chat.
4. Family members chat frequently.
5. Family members are always talking about future.
6. Parents demand obedience from children.
7. Parents make family decisions.
8. Parents angry over disagreements.
9. Parents demand compliance with rules.
10. Parents always say, "when you grow up you will understand".
11. After learning about the pandemic, the frequency of discussing the following topics with your family members (parents/adult children) is:

*(Measured with a 5-point Likert scale ranging from 1= never to 5= most frequently)*

1. I share information and knowledge related to COVID-19 with family members (e.g., infection, transmission).
2. I share news regarding the severity of COVID-19 with family members.
3. I share news regarding treatment with family members.
4. My family and I discuss the scope of the pandemic.
5. My family and I talk about the consequences of infection.
6. My family and I discuss about the measure of social distancing.
7. My family and I discuss the measure of wearing a mask.
8. My family and I discuss the measure of washing hands regularly.
9. My family and I had disagreements over COVID-19 prevention.
10. My family and I had disagreements over COVID-19 treatments.
11. What is your gender? (Male; female)
12. What is your age?
13. What is your highest level of education?
14. What is your average monthly income?
